# One-photon scattering by an atomic chain in a two-mode resonator: cyclic conditions

**DOI:** 10.1186/1556-276X-9-203

**Published:** 2014-05-01

**Authors:** Andrii S Sizhuk, Stanislav M Yezhov

**Affiliations:** 1 Department of Radiophysics, Kyiv Taras Shevchenko National University, Acad. Glushkova Avenue 4-g, Kyiv 03022, Ukraine; 2 Department of Physics, Kyiv Taras Shevchenko National University, Acad. Glushkova Avenue 2, Kyiv 03022, Ukraine

**Keywords:** Atoms, Fock state, Weisskopf-Wigner approximation

## Abstract

In this work, a chain of *N* identical two-level atoms coupled with a quantized electromagnetic field, initially prepared via a single-photon Fock state, is investigated. The *N*-particle state amplitude of the system is calculated for several space configurations of the atoms in the Weisskopf-Wigner approximation. It was shown that the space configuration of an atomic chain, the total number of atoms, and even the available volume for the field modes define the behavior of the system state amplitude with time. Applying the condition of ‘cyclic bonds’, presented in this work, to the elaborated theory allows to describe the system time evolution, practically, for any space configuration.

## Background

The collective absorption (emission) of photons by an ensemble of identical atoms ‘provides valuable insights into the many-body physics of photons and atoms’ (quoted from [1]). Taking into account the quantization of electromagnetic field, many fundamental and interesting properties of the coupled systems of atoms and field are revealed. For example, when the average distances between atoms are much less than the ‘resonant transition’ wavelength of emitted (absorbed) light, the cooperative coupling leads to a substantial radiative shift of the transition energy and significant change in decay rate of the ensemble state. The latter was revealed through the various theoretical (for example, some relatively modern researches in [2-5]) and experimental investigations (see starting, for example, from [6,7] to the modern applications like described in [8] and impressively effective experimental realizations as in [1]). Some peculiar behavior in spontaneous emission is proper even in a system of atoms which can have a relative distance larger than the emission wavelength (see, for instance, [9]), and initially, only one atom or one-photon state is excited, as discovered in this paper.

In the present paper, a system (chain) of *N* identical two-level non-interacting atoms, prepared ‘via a single-photon Fock state’ in the one- or two-mode resonator, is investigated. The main goal of the paper is to obtain the information about the state of electromagnetic field and atomic system (chain) in a Weisskopf-Wigner approximation (see [10] chapter 6, page 206 and some comments in [11]). The calculations of the state amplitudes of the atomic system are made for several approximations in resonator (cavity) characteristics and for several types of space configurations of the atoms in the chain.

In this work, we study the case, in which the distances between atoms are quite large, so that the average distances between atoms are greater or in the same order than the ‘resonant transition’ wavelength. Therefore, we prepare an ensemble of *N* two-level atoms initially in ground state, and a single mode of the radiation field is excited in a ‘Fock’ state (so called one-photon state). This is the case of a purely monochromatic wave with zero line width under the consideration. A laser output in single mode operation can approximate this situation due to its high degree of monochromaticity (small line width) for instance. The mode of electromagnetic field is specified completely by giving its wave vectors **k**_0_ with atomic transition frequency *ω *= *c*|**k**_0_| and its polarization *j* (*j *= 1, 2).

The main feature, differentiating our research from others in this domain, is the developed direct and consistent solution to the *N*-particle equations, describing the time evolution of the *N* atomic probability state amplitudes. Besides, in certain sense, we explained the nature of the widely used Weisskopf-Wigner approximation that was not found in the reviewed by us scientific literature.

The goal of this paper can be formulated as an attempt to propose an adapted and simple in practical use theory, for example in the highly applied nanoscale physics. The proposed theoretical material requires corresponding experimental verification. As an idea of an application, the model system can be realized on atomic (developing the method proposed in [1] for the nuclei of ^57^Fe in certain composites, but this time for a visible region), chains of trapped ions (like in [8]), and molecular structures for further developing such techniques like FRET (described for instance in [12]), atomic chains like carbyne loops (for example, [13]), and microhole array synthesized by femtosecond laser radiation (see [14], for an instance).

Let us first provide below some general theoretical premises. More detailed derivations of the corresponding mathematical model can be found in [11].

## Methods

### The equations of motion for the state amplitudes

We have assumed that the atomic energy levels have no linewidth, so that, only if $\omega =\omega_{k_{0}}=\Delta E_{\text{ab}}/\hbar$, the atoms can be able to absorb a photon. Obviously, this is an unrealistic case since it is impossible to have a completely monochromatic wave. In addition, for the case of the Fock initial state, in which we measured the energy precisely of the mode, the average electric field will be zero. In the forth of the law of energy conservation, an emitted photon will correspond to the same frequency $\omega_{k_{0}}$ (we can say it will occur with a high probability after a quite long time interval if the system has a damping).

Therefore, consider a collection of *N* identical atoms, at positions **r**_1_,…,**r**_*α*_,…,**r**_*N*_, coupled to a one mode electromagnetic (EM) field. Each atom *α *= 1..*N *is assumed to have only the two states |*a*〉_*α *_and |*b*〉_*α*_, separated by energy $E_{\alpha }=E_{a\alpha }-E_{b\alpha }=\hbar \omega$. In the dipole approximation, the closed conservative system of identical atoms with the electromagnetic field in a cavity can be described by the Hamiltonian consisting of free atoms and electromagnetic field items with dipole-field coupling between the atoms and the electromagnetic field modes.

Inasmuch as at the initial time moment *t *= 0 all atoms *α *= 1..*N* of the ensemble are in the ground state |*b*〉_*α*_ and EM field is in Fock state $|1_{k_{0}}\rangle$ (that presents one photon with the wave vector **k**_0_), we look for a solution of the corresponding Schrödinger equation in the interacting picture in the following form:

(1) $$\begin{array}{ll} \Psi = & \overset{N}{\underset{\alpha =1}{\sum }}\beta_{\alpha }(t)|b_{1}b_{2}\ldots a_{\alpha }\ldots b_{N}\;0\rangle \\ & +\underset{k,j}{\sum }\gamma_{k,j}(t)|b_{1}b_{2}\ldots b_{N}1_{k,j}\rangle \end{array}$$

with the initial conditions:

(2) $$\beta_{\alpha }(0)=0,\;\gamma_{k,j}(0)=\delta_{k,k_{0}},$$

where $\delta_{k,k_{0}}$ is Kroneker’s delta symbol. $\delta_{k,k_{0}}=1$ if **k **= **k**_0_, and $\delta_{k,k_{0}}=0$ if **k **≠ **k**_0_. *β*_*α *_(*t*) (*α *= 1..*N*) and *γ*_**k**,*j *_(*t*) (*j *= 1, 2) are the *α*th atom excited state amplitude with the others in the ground states and excited Fock field state amplitude of the *j*th polarization with the wave vector **k**, accordingly.

Then, the corresponding Schrödinger equation in the interacting picture yields the following system of equations:

(3) $${\dot{\beta }}_{\alpha }(t)=i\underset{k,j}{\sum }g_{\alpha }^{*}(k,j)\gamma_{k,j}(t)\text{exp}(-i(\nu_{k}-\omega )t+i{\mathrm{kr}}_{\alpha });$$

(4) $${\dot{\gamma }}_{k,j}(t)=i\overset{N}{\underset{\delta =1}{\sum }}g_{\delta }(k,j)\beta_{\delta }(t)\text{exp}(i(\nu_{k}-\omega )t-i{\mathrm{kr}}_{\delta }),$$

where

(5) $$g_{\alpha }(k,j)=\sqrt{\frac{\nu_{k}}{2\hbar \varepsilon_{0}V}}\;{℘}_{\alpha }\cdot e_{k,j},$$

where

(6) $${℘}_{\alpha }\cdot e_{k,j}=|{℘}_{\alpha }|\text{cos}\theta_{k,j.}$$

Here, *θ*_**k**,*j *_is the angle between dipole transition vector *℘*_*α *_(more accurately, non-diagonal dipole matrix element) and the *j*th unit polarization vector **e**_**k**,*j*_ (*j *= 1,2 and **e**_**k**,*j *_· **k**= 0). *V *is the available by the system of atoms and field space volume. The frequencies *ν*_*k *_correspond to the modes with the module of the wave vectors *k *equal to |**k**|.

Therefore, substituting Equation 4 into 3 and differentiating one more time, after applying the Weisskopf-Wigner approximation (details in [11]), we can derive the following system of evolution equations:

(7) $$\frac{d^{2}}{dt^{2}}\beta_{\alpha }(t)=-\overset{N}{\underset{\delta =1}{\sum }}\beta_{\delta }(t)\Phi_{\alpha \delta }-2D_{\alpha }\frac{d}{\text{dt}}\beta_{\alpha }(t),$$

where

(8) $$\Phi_{\alpha \delta }=\underset{j,\;|k|=k_{0}}{\sum }g_{\alpha }^{*}(k,j)g_{\delta }(k,j)\text{exp}[ik(r_{\alpha }-r_{\delta })].$$

And the decay rates *D*_*α *_in the approximation can be estimated by the formula:

(9) $$\;D_{\alpha }=\frac{1}{2}\frac{8\pi }{3}\frac{2\pi }{2\hbar \varepsilon_{0}}{\left(\frac{1}{2\pi \;c}\right)}^{3}\;|℘{|}^{2}\omega^{3}=\frac{1}{2}\frac{1}{3\pi \;\hbar \varepsilon_{0}c^{3}}\;|℘{|}^{2}\omega^{3}.$$

The coefficient *D*_*α *_(*α *= 1..*N*) describes the respective rate of decay for *α*th atom excited state. Note, that the ‘non-resonant’ items for the particle with distinguished from *α *indexes were disregarded in here in an assumption of quite large interatomic distances (see details in [11]).

## Results and discussion

### An atomic chain with cyclically distanced atoms

$$\overset{N}{\underset{\alpha }{\sum }}\text{sin}({\text{kr}}_{\alpha })\text{cos}({\text{kr}}_{\alpha })=0$$

Next, we try to make the calculations, using here the particular case of space configuration for the system atoms field. Below, for simplicity, only one polarized mode (*j* = 1) of the resonant field modes is taken into account with the common parameters *g*_*α *_and *℘*_*α *_for *α *= 1..*N *:

(10) $$g_{\alpha }(k,j)=g_{\alpha }=g>0$$

and

(11) $${℘}_{\alpha }\cdot e_{k,j}=|℘|$$

for |**k**| = *k*_0_. In other words, the space angle distribution for the components *Φ*_*αδ*_is disregarded here, assuming the direction of the transition dipole moment *℘*_*α *_for any atom in the system coincides with the photon polarization in absorbing or emitting a resonant photon.

Then, from the system of Equation 7, in the case of a cavity with two resonant modes **k **= ± **k**_0 _and identical atoms with *D*_*α *_≡ *D *for *α *= 1..*N*, one derives that

(12) $$\begin{array}{l} \frac{d^{2}}{dt^{2}}\beta_{\alpha }(t)=-2g^{2}\overset{N}{\underset{\delta =1}{\sum }}\beta_{\delta }(t)\text{cos}(k(r_{\alpha }-r_{\delta }))-2D\frac{d}{\text{dt}}\beta_{\alpha }(t). \end{array}$$

Using the notation

(13) $$B_{c}(t)=\overset{N}{\underset{\alpha =1}{\sum }}\beta_{\alpha }(t)\text{cos}\left({\mathrm{kr}}_{\alpha }\right)$$

and the ‘cyclic’ condition $\overset{N}{\underset{\alpha }{\sum }}\text{sin}({\mathrm{kr}}_{\alpha })\text{cos}({\mathrm{kr}}_{\alpha })=0$ yields the following relatively simple linear differential equation:

(14) $$\frac{d^{2}}{dt^{2}}B_{c}(t)=-2g^{2}\underset{\alpha }{\sum }\overset{2}{\text{cos}}({\mathrm{kr}}_{\alpha })B_{c}(t)-2D\frac{d}{\text{dt}}B_{c}(t).$$

Therefore, taking into account the initial conditions *β*_*δ *_(0) = 0 for *α *= 1..*N*, the solution of the above equation is as follows:

(15) $$\;B_{c}=\underset{\alpha }{\sum }\beta_{\alpha }(t)\text{cos}({\mathrm{kr}}_{\alpha })=C\left(\text{exp}\left(\Omega_{2+}t\right)-\text{exp}\left(\Omega_{2-}t\right)\right),$$

where $\Omega_{2}=g\sqrt{2\underset{\alpha }{\sum }\overset{2}{\text{cos}}({\mathrm{kr}}_{\alpha })}$ and

(16) $$\Omega_{2\pm }=-D\pm \sqrt{D^{2}-\Omega_{2}^{2}}.$$

By analogy,

(17) $$\;B_{s}=\underset{\alpha }{\sum }\beta_{\alpha }(t)\text{sin}({\mathrm{kr}}_{\alpha })=C^{\prime }\left(\;\text{exp}\left(\;\overset{\prime }{\underset{2+}{\Omega }}t\;\right)-\text{exp}\left(\;\overset{\prime }{\underset{2-}{\Omega }}t\;\right)\;\right),$$

where $\Omega_{2}^{\prime }=g\sqrt{2\underset{\alpha }{\sum }\overset{2}{\text{sin}}({\mathrm{kr}}_{\alpha })}$ and

(18) $$\Omega_{2\pm }^{\prime }=-D\pm \sqrt{D^{2}-{\Omega^{\prime }}_{2}^{2}}.$$

It is easy to see, that ${\Omega^{\prime }}_{2}^{2}+\Omega_{2}^{2}=2g^{2}N$.

The field probability amplitudes can be obtained using the subsystem of Equation 4 of the full ‘conservative’ system of Equations 3 and 4. Therefore, substituting (15) and (17) into the Equation 4, and then taking into account the restrictions *β*_*α *_(0) = 0 for *α *= 1..*N*, we obtain that

(19) $$\begin{array}{l} \gamma_{k}(t)=2\text{ig}\left[C\left\{\Omega_{2+}f\left(\Omega_{2+},t\right)-\Omega_{2-}f\left(\Omega_{2-},t\right)\right\}\right)\\ \left(-iC^{\prime }\left\{\Omega_{2+}^{\prime }f\left(\Omega_{2+}^{\prime },t\right)-\Omega_{2-}^{\prime }f\left(\Omega_{2-}^{\prime },t\right)\right\}\right]+1; \end{array}$$

and

(20) $$\begin{array}{l} \gamma_{-k}(t)=2\text{ig}\left[C\left\{\Omega_{2+}f\left(\Omega_{2+},t\right)-\Omega_{2-}f\left(\Omega_{2-},t\right)\right\}\right)\\ \left(+\;iC^{\prime }\left\{\Omega_{2+}^{\prime }f\left(\Omega_{2+}^{\prime },t\right)-\Omega_{2-}^{\prime }f\left(\Omega_{2-}^{\prime },t\right)\right\}\right]. \end{array}$$

where

(21) $$f\left(\Omega ,t\right)=\frac{\text{exp}\left(\frac{\Omega t}{2}\right)}{\Omega^{2}}\text{sinh}\left(\frac{\mathrm{\Omega t}}{2}\right).$$

Note, here, we neglected the possible space angle distribution for the direction of the resonant wave vector **k**.

Inasmuch as cos(**k (r**_*α *_- **r**_*δ*_**)**) = cos (**kr**_*α*_) cos (**kr**_*δ*_) + sin (**kr**_*α*_) sin (**kr**_*δ*_), then, after substitution of the found superpositions (15) and (17) into the initial Equation 12, we derive the following integrable differential equation:

(22) $$\begin{array}{ll} \frac{d^{2}}{dt^{2}}\beta_{\alpha }(t)+2D\frac{d}{\text{dt}}\beta_{\alpha }(t)= & -2g^{2}\left\{\text{cos}\left({\mathrm{kr}}_{\alpha }\right)B_{c}(t)\right)\\ & \left(+\text{sin}\left({\mathrm{kr}}_{\alpha }\right)B_{s}(t)\right\}. \end{array}$$

Integrating the left and right sides of the equation above (22) over time yields

(23) $$\frac{d}{\text{dt}}\beta_{\alpha }(t)+2D\beta_{\alpha }(t)=T_{\alpha }(t),$$

where

(24) $$\begin{array}{ll} T_{\alpha }(t)= & -2g^{2}\left\{\text{cos}\left({\mathrm{kr}}_{\alpha }\right)F_{c}(t)+\text{sin}\left({\mathrm{kr}}_{\alpha }\right)F_{s}(t)\right\}\\ & +\frac{d}{\text{dt}}\beta_{\alpha }(0)+2D\beta_{\alpha }(0), \end{array}$$

and

(25) $$F_{c,s}(t)=\overset{t}{\underset{0}{\int }}\left[B_{c,s}(t)\right]\mathrm{dt}.$$

According to the definition of the functions *F*_*c*,*s *_(*t*)

(26) $$\begin{array}{l} \;F_{c}(t)=C\left\{\frac{1}{\Omega_{2+}}\left[\text{exp}\left(\Omega_{2+}t\right)-1\right]-\frac{1}{\Omega_{2-}}\left[\text{exp}\left(\Omega_{2-}t\right)-1\right]\right\}; \end{array}$$

and

(27) $$\begin{array}{l} \;F_{s}(t)=C^{\prime }\left\{\;\frac{1}{\Omega_{2+}^{\prime }}\left[\;\text{exp}\left(\;\overset{\prime }{\underset{2+}{\Omega }}t\;\right)-1\;\right]-\frac{1}{\Omega_{2-}^{\prime }}\left[\;\text{exp}\left(\;\overset{\prime }{\underset{2-}{\Omega }}t\;\right)-1\;\right]\;\right\}. \end{array}$$

The solution of such linear first order differential equation, like (23), has the form:

(28) $$\beta_{\alpha }(t)=\frac{1}{\text{exp}\left(2\text{Dt}\right)}\int \left[T_{\alpha }(t)\text{exp}\left(2\text{Dt}\right)\right]\mathrm{dt}.$$

The integration in the last expression can be performed, yielding

(29) $$\begin{array}{ll} \int & \left[T_{\alpha }(t)e^{\left(2\text{Dt}\right)}\right]\text{dt}\\ = & -2g^{2}\left\{\text{cos}\left({\mathrm{kr}}_{\alpha }\right)C\left\{\frac{1}{\Omega_{2+}}\left[\frac{1}{\Omega_{2+}+2D}\left(e^{\left((2D+\Omega_{2+})t\right)}-1\right)\right)\right)\right)\\ & \left(-\frac{1}{2D}\left(e^{\left(2\text{Dt}\right)}-1\right)\right]\left(-\frac{1}{\Omega_{2-}}\left[\frac{1}{\Omega_{2-}+2D}\left(e^{\left((2D+\Omega_{2-})t\right)}-1\right)\right)\right)\\ & \left(\left(-\frac{1}{2D}\left(e^{\left(2\text{Dt}\right)}-1\right)\right]\right\}+\text{sin}\left({\mathrm{kr}}_{\alpha }\right)C^{\prime }\\ & \left(\times \left\{\frac{1}{\Omega_{2+}^{\prime }}\left[\frac{1}{\Omega_{2+}^{\prime }+2D}\left(e^{\left((2D+\Omega_{2+}^{\prime })t\right)}-1\right)\right)\right)\right)\\ & \left(-\frac{1}{2D}\left(e^{\left(2\text{Dt}\right)}-1\right)\right]-\left(\frac{1}{\Omega_{2-}^{\prime }}\left[\;\frac{1}{\Omega_{2-}^{\prime }+2D}\left(\;e^{\left(\;(2D+\Omega_{2-}^{\prime })t\;\right)}-1\;\right)\right)\right)\\ & \;\left(\left(\left(-\;\frac{1}{2D}\left(e^{\left(2\text{Dt}\right)}-1\right)\right]\right\}\right\}\\ & +\left(\frac{d}{\text{dt}}\beta_{\alpha }(0)+2D\beta_{\alpha }(0)\right)\frac{1}{2D}\left(e^{\left(2\text{Dt}\right)}-1\right)+C_{0}. \end{array}$$

Therefore,

(30) $$\begin{array}{l} \beta_{\alpha }(t)=\\ =-2g^{2}\left\{\text{cos}\left({\mathrm{kr}}_{\alpha }\right)C\left[H\left(\Omega_{2+},D,t\right)-H\left(\Omega_{2-},D,t\right)\right]\right)\\ \left(+\text{sin}\left({\mathrm{kr}}_{\alpha }\right)C^{\prime }\left[H\left(\Omega_{2+}^{\prime },D,t\right)-H\left(\Omega_{2-}^{\prime },D,t\right)\right]\right\}\\ +\left(\frac{d}{\text{dt}}\beta_{\alpha }(0)+2D\beta_{\alpha }(0)\right)\frac{1}{2D}\left(1-e^{-\left(2\text{Dt}\right)}\right)+C_{0}e^{-\left(2\text{Dt}\right)}, \end{array}$$

where

(31) $$\begin{array}{ll} H\left(\Omega ,D,t\right)= & \frac{1}{\Omega }\left[\frac{1}{\Omega +2D}\left(e^{\left(\mathrm{\Omega t}\right)}-e^{-\left(2\text{Dt}\right)}\right)\right)\\ & \left(-\frac{1}{2D}\left(1-e^{-\left(2\text{Dt}\right)}\right)\right]. \end{array}$$

The initial condition *β*_*α *_(0) = 0, for *α *= 1..*N*, sets the coefficient *C*_0 _equals 0. The initial time derivative $\frac{d}{\text{dt}}\beta_{\alpha }(0)$ can be determined, for example, if the system of Equation 3 from the initial ‘conservative’ full system of Equations 3 and 4 is chosen as a basis at the time moment *t *= 0. Then, the initial condition for the field state amplitude *γ*_**k **_(0) = 1, where **k **= **k**_0_, sets the time derivative $\frac{d}{\text{dt}}\beta_{\alpha }(0)$ to the following expression:

(32) $$\begin{array}{l} \frac{d}{\text{dt}}\beta_{\alpha }(0)=\text{ig}\left\{e^{{\mathrm{kr}}_{\alpha }}\gamma_{k}(0)+e^{{-\text{kr}}_{\alpha }}\gamma_{-k}(0)\right\}\\ =\text{ig}\left\{\text{cos}\left({\mathrm{kr}}_{\alpha }\right)+i\text{sin}\left({\mathrm{kr}}_{\alpha }\right)\right\}. \end{array}$$

Now, the question arises how to choose correctly the coefficients *C *and *C*^′^. First of all, the choice has to satisfy the limitations on the probability amplitude, yielding the corresponding probability limited above by unit (the sum of all the modules squared of the introduced amplitudes equals unit probability). Secondly, the solution with the coefficients have to be consistent with the model decay (damping).

We observe that, formally, when the real part of the variable *Ω* is a negative quantity, that is *Re *(*Ω*) < 0, the introduced functions *H *and *f * have the following limits for quite long time intervals:

(33) $$\underset{t\to \infty }{\text{lim}}H\left(\Omega ,D,t\right)=-\frac{1}{2D\Omega },\;\text{when}\;\text{Re}\left(\Omega \right)<0;$$

(34) $$\underset{t\to \infty }{\text{lim}}f\left(\Omega ,t\right)=-\frac{1}{2\Omega^{2}},\;\text{when}\;\text{Re}\left(\Omega \right)<0.$$

Then,

(35) $$\begin{array}{l} \;\underset{t\to \infty }{\text{lim}}\gamma_{k}(t)=\text{ig}\left[\;C\left\{\;\frac{1}{\Omega_{2-}}-\frac{1}{\Omega_{2+}}\;\right\}-iC^{\prime }\left\{\;\frac{1}{\Omega_{2-}^{\prime }}-\frac{1}{\Omega_{2+}^{\prime }}\;\right\}\;\right]+1; \end{array}$$

(36) $$\begin{array}{l} \;\underset{t\to \infty }{\text{lim}}\gamma_{-k}(t)=\text{ig}\left[C\left\{\frac{1}{\Omega_{2-}}-\frac{1}{\Omega_{2+}}\right\}+iC^{\prime }\left\{\frac{1}{\Omega_{2-}^{\prime }}-\frac{1}{\Omega_{2+}^{\prime }}\right\}\right]; \end{array}$$

(37) $$\begin{array}{l} \underset{t\to \infty }{\text{lim}}\beta_{\alpha }(t)=-\frac{1}{D}g^{2}\left\{C\left\{\frac{1}{\Omega_{2-}}-\frac{1}{\Omega_{2+}}\right\}\text{cos}\left({\mathrm{kr}}_{\alpha }\right)\right)\\ \left(+C^{\prime }\left\{\frac{1}{\Omega_{2-}^{\prime }}-\frac{1}{\Omega_{2+}^{\prime }}\right\}\text{sin}\left({\mathrm{kr}}_{\alpha }\right)\right\}+\frac{1}{2D}\frac{d}{\text{dt}}\beta_{\alpha }(0). \end{array}$$

As for an open system, in our case, it should be expected for a quite long time interval the total electromagnetic energy of the atoms-field system to be emitted into the subsystem causing the state damping. Therefore, let us define the coefficients *C *and *C*^′ ^in the following manner:

(38) $$C=\frac{i\Omega_{2-}\Omega_{2+}}{2g\left(\Omega_{2+}-\Omega_{2-}\right)}$$

and

(39) $$C^{\prime }=-\frac{\Omega_{2-}^{\prime }\Omega_{2+}^{\prime }}{2g\left(\Omega_{2+}^{\prime }-\Omega_{2-}^{\prime }\right)}.$$

Then, after substitution into the expressions for the time limits, one derive the logical finale of the system evolution:

(40) $$\underset{t\to \infty }{\text{lim}}\gamma_{k}(t)=0;$$

(41) $$\underset{t\to \infty }{\text{lim}}\gamma_{-k}(t)=0;$$

(42) $$\begin{array}{l} \underset{t\to \infty }{\text{lim}}\beta_{\alpha }(t)=0. \end{array}$$

The possible space configurations of the atomic system, satisfying the condition of ‘circularity’, can be easily found. For example, the set s3a1 (the notation ‘s3a1’ is just introduced here): ${\mathrm{kr}}_{1}\equiv k\cdot r_{1}=\frac{\pi }{6}$, ${\mathrm{kr}}_{2}=\frac{2\pi }{3}$, and **kr**_3 _= *π*. As an instance, it can also be the set s3a2: ${\mathrm{kr}}_{1}=\frac{2\pi }{3}$, ${\mathrm{kr}}_{2}=\frac{3\pi }{2}$, and ${\mathrm{kr}}_{3}=\frac{\pi }{6}+2\pi$. A space configuration for five atoms can be represented, for example, by such set like s5a1: ${\mathrm{kr}}_{1}\equiv k\cdot r_{1}=\frac{2\pi }{3}$, ${\mathrm{kr}}_{2}=\frac{3\pi }{2}$, ${\mathrm{kr}}_{3}=\frac{5\pi }{2}$, ${\mathrm{kr}}_{4}=\frac{7\pi }{2}$, and ${\mathrm{kr}}_{5}=\frac{19\pi }{6}$. Specifically, a combination, such like ${\mathrm{kr}}_{i}=\pm \;\frac{\pi }{n}+o_{i}\left(\frac{\pi }{n}\right)+2i\pi$ with *i *= 1..*N*, *n *> 2, and $\left|o_{i}\left(\frac{\pi }{n}\right)\right|<<\frac{\pi }{n}$, can generate the necessary magnitudes of the characteristic system frequencies *Ω*_2 _and $\Omega_{2}^{\prime }$ (that, actually, are the corresponding Rabi frequencies), comparable with the given magnitude of the decay coefficient *D*.

Below we depict the atomic system behavior in the several introduced above configurations. Note, that the cited thereby Rabi frequencies were calculated in the SI system of units with the following notations: $\hbar \approx 1.05457\times 10^{-34}\;\text{J sec/rad}$; the electric permittivity of free space *ε*_0 _≈ 8.8542 × 10^-12^ F/m; the speed of light in free space *c *= 299792458 m/sec; resonant wavelength close to the *D*_2_-line of a sodium atom *λ*_*D *_≈ 589.29 × 10^-9^ m; corresponding circular (in radians per second) resonant frequency $\omega_{\text{res}}=\frac{2\pi c}{\lambda_{D}}\approx 0.101747\times 10^{16}\pi \;\text{rad/sec}$; non-diagonal so called ‘transition’ dipole matrix element (in the same order as for the *D*_2_-line transition, that is about 1 Debye) *ρ*_ex _= 1 × 3.33564 × 10^-30 ^C m. For instance, if the available for the system of atoms and field volume has the value equal to *V *= 0.001 m^3^, then $g=\rho_{\text{ex}}\sqrt{\frac{\omega_{\text{res}}}{2\hbar \varepsilon_{0}V}}\approx 77.8597\sqrt{\pi }\;\text{rad/sec}$.

**Figure 1:**
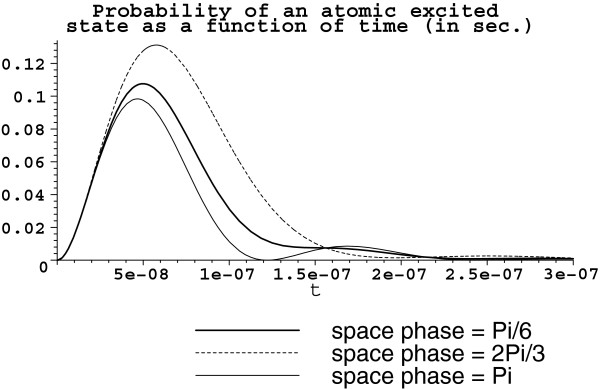
**Time evolution of |*β***_***α ***_**(*t*)|**^**2**^***. V *= 10**^**-13 **^**m**^**3**^**. **Atoms are arranged in the set s3a1 with *D *≈ 10^7^ rad/sec. The bold solid line represents the atom with the space phase **kr**_1 _= *π*/6, the dot line is for the space phase **kr**_2 _= 2*π*/3, and the thin solid line corresponds to **kr**_3 _= *π*.

Assume, for example, the available volume *V *= 10^-13^ m^3 ^is somehow filled by the set s3a1 with *D *≈ 10^7^ rad/sec, initially coupled with one-photon Fock state. Then, $g=\rho_{\text{ex}}\sqrt{\frac{\omega_{\text{res}}}{2\hbar \varepsilon_{0}V}}\approx 77.8597\times 10^{5}\sqrt{\pi }\;\text{rad/sec}$, $\Omega_{2}\approx 155.7195\times 10^{5}\sqrt{\pi }\;\text{rad/sec}$, and $\Omega_{2}^{\prime }\approx 77.8597\times 10^{5}\sqrt{2\pi }\;\text{rad/sec}$. The corresponding graphs for probability to find each atom in the excited state are shown in Figure 1.

**Figure 2:**
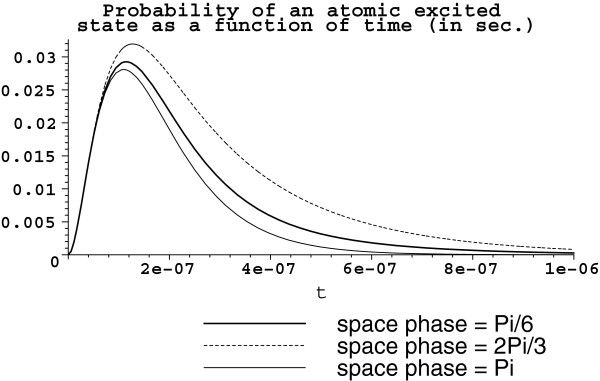
**Atom excited state probability |*β***_***α ***_**(*t*)|**^**2**^***. V *= 10**^**-12 **^**m**^**3**^**. **Atoms are arranged in the set s3a1 with *D *≈ 10^7^ rad/sec. The bold solid line represents the atom with the space phase **kr**_1 _= *π*/6, the dot line is for the space phase **kr**_2 _= 2*π*/3, and the thin solid line corresponds to **kr**_3 _= *π*.

Let us see what happens when the available volume is increased by one order. This yields *V *= 10^-12 ^m^3 ^with the same three atoms (*D *≈ 10^7 ^rad/sec) of the configuration s3a1. Then, $g\approx 24.6214\times 10^{5}\sqrt{\pi }\;\text{rad/sec}$; $\Omega_{2}\approx 49.2428\times 10^{5}\sqrt{\pi }\;\text{rad/sec}$ and $\Omega_{2}^{\prime }\approx 24.6214\times 10^{5}\sqrt{2\pi }\;\text{rad/sec}$. The corresponding graphs for each atom excited state probability are depicted in Figure 2.

**Figure 3:**
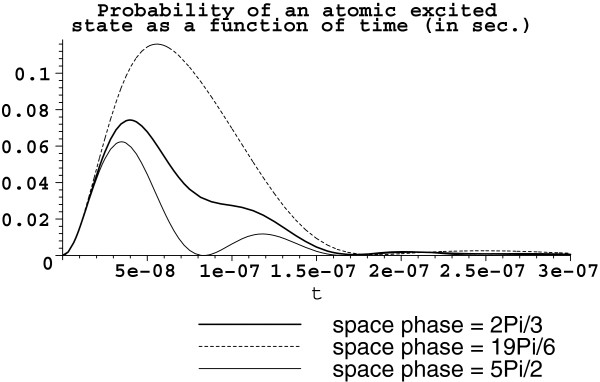
**Atomic excitation probability |*β***_***α ***_**(*t*)|**^**2 **^**as a function of time. ***V *= 10^-13 ^m^3^. Atoms are arranged in the set s5a1 with *D *≈ 10^7 ^rad/sec. The bold solid line represents the atom with the space phase **kr**_1 _= 2*π*/3, the dot line is for the space phase **kr**_5 _= 19*π*/6, and the thin solid line corresponds to **kr**_3 _= 5*π*/2.

Suppose now that the available volume is *V *= 10^-13 ^m^3^, somehow filled by the set s5a1 with *D *≈ 10^7 ^rad/sec initially coupled with one-photon Fock state. Then, $g=\rho_{\text{ex}}\sqrt{\frac{\omega_{\text{res}}}{2\hbar \varepsilon_{0}V}}\approx 77.8597\times 10^{5}\sqrt{\pi }\;\text{rad/sec}$; $\Omega_{2}\approx 77.8597\times 10^{5}\sqrt{2\pi }\;\text{rad/sec}$, and $\Omega_{2}^{\prime }\approx 155.7195\times 10^{5}\sqrt{2\pi }\;\text{rad/sec}$. The corresponding graphs for each atom excited state probability are shown in Figure 3.

**Figure 4:**
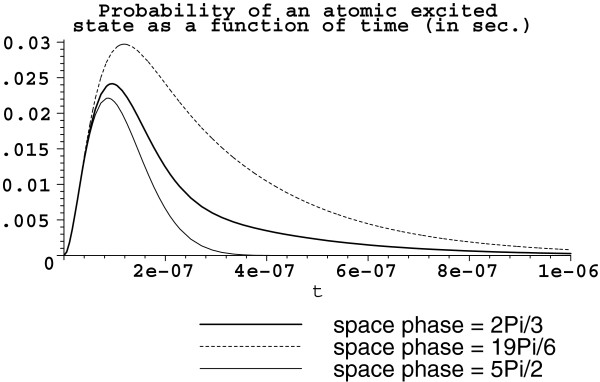
**Probability |*β***_***α ***_**(*t*)|**^**2**^**. ***V *= 10^-12 ^m^3^. Atoms are arranged in the set s5a1 with *D *≈ 10^7 ^rad/sec. The bold solid line represents the atom with the space phase **kr**_1_= 2*π*/3, the dot line is for the space phase **kr**_5 _= 19*π*/6, and the thin solid line corresponds to **kr**_3 _= 5*π*/2.

And again, let us see what happens when the available volume is increased by one order. This yields *V *= 10^-12 ^m^3 ^with the same five atoms (*D* ≈ 10^7 ^rad/sec) of the configuration s5a1. Then, $g\approx 24.6214\times 10^{5}\sqrt{\pi }\;\text{rad/sec}$; $\Omega_{2}\approx 24.6214\times 10^{5}\sqrt{2\pi }\;\text{rad/sec}$, and $\Omega_{2}^{\prime }\approx 49.2428\times 10^{5}\sqrt{2\pi }\;\text{rad/sec}$. The corresponding graph is in Figure 4. Note that the graphs Figures 3 and 4 of excited state probabilities are for the chosen three atoms with the following phases: ${\mathrm{kr}}_{1}=\frac{2\pi }{3}$, ${\mathrm{kr}}_{3}=\frac{5\pi }{2}$, and ${\mathrm{kr}}_{5}=\frac{19\pi }{6}$.

As it was supposed in the derivative of the differential equations with the damping items such like (12) (see the details in the work [11], the available volume *V *for the system of atoms and field defines the ‘available’ modes for the electromagnetic field. The value of volume *V* can determine one of the inequalities *D *< *Ω*_2 _and *D *> *Ω*_2 _($D<\Omega_{2}^{\prime }$ and $D>\Omega_{2}^{\prime }$), therefore defining the character of the system relaxation. Such fundamental system property was illustrated in the figures. It is interesting to note that increasing the system volume *V*, therefore increases the ‘available’ number of quantized field modes, the maximum probability to find an atom in its excited state decreases. Other interesting feature, shown in the proposed graphs, is the different character of relaxation for each excited atom. The latter depends, as shown here, on the space phase **kr**_*α*_, where *α *= 1..*N*. 

On this note, therefore, let our narration to come to the following conclusions, in short.

## Conclusions

Thus, in this work, we investigated a chain of *N* identical two-level long distanced atoms prepared ‘via a single-photon Fock state’. The functional dependence of the atomic state amplitudes on a space configuration and time is derived in the Weiskopf-Wiegner approximation.

It was shown that in increasing the system volume *V*, the maximum value of probability to find an atom in its excited state decreases. The feature can be experimentally investigated at the proposed nanoscale limit for the space configuration of atoms.

Hence, the Weiskopf-Wiegner approximation was revealed through the provided application to the many-body system at the nanoscale limit for the atomic space phases. The found solution (30) cannot be counted as a particular one, or as a limit of such, for the initial systems of Equations 3 and 4 that represent only a closed conservative system of atoms and an electromagnetic field. Thus, we can say that the model described in this work, besides the atoms and the electromagnetic field, implicitly contains a third participant guaranteeing a total system relaxation with time. It is interesting to note here that the ‘complete’ decay of the system excitations was strongly imposed by the choice of the coefficients *C* (38) and *C*^′^ (39).

The methods, described in this work, of solving the system of linear differential equations can be applied even for more general situations when the boundary ‘circular’ conditions are not satisfied. In certain cases, the problem allows to extend the system by adding a subsystem of non-sufficient number of atoms (or an atom when the expression $\left|\overset{N}{\underset{\alpha }{\sum }}\text{sin}({\mathrm{kr}}_{\alpha })\text{cos}({\mathrm{kr}}_{\alpha })\right|<1$ is actual) without influencing the main characteristics under our interest. Besides, it is interesting to investigate the dependence of value of the mentioned construction $\left|\overset{N}{\underset{\alpha }{\sum }}\text{sin}({\mathrm{kr}}_{\alpha })\text{cos}({\mathrm{kr}}_{\alpha })\right|$ on the location of the origin of a coordinate system.

## Competing interests

The authors declare that they have no competing interests.

## Authors’ contributions

SAS performed the calculations and analysis of the results. YSM analyzed the methods of investigation and drafted the manuscript. Both authors read and approved the final manuscript.
